# Benzodiazepine Addiction and Suicidal Risk in the Elderly

**DOI:** 10.1192/j.eurpsy.2026.11590

**Published:** 2026-07-31

**Authors:** N. Smaoui, M. Barkallah, N. Charfi, R. Feki, M. Maalej, I. Gassara, S. Omri, L. Zouari

**Affiliations:** Psychiatry Department “C”, Hedi Chaker Hospital, Sfax, Tunisia

## Abstract

**Introduction:**

Prolonged benzodiazepine (BZD) use in elderly individuals has been associated with an increased risk of suicide.

**Objectives:**

This study aimed to investigate the relationship between BZD addiction and suicidal risk in older adults.

**Methods:**

A cross-sectional study was conducted among patients aged 65 years and older attending outpatient psychiatric consultations. Data collected included benzodiazepine use and detailed assessment of suicidal history. Suicidal risk was assessed using the Ducher Suicidal Risk Scale, and benzodiazepine dependence was evaluated with the Benzodiazepine Attachment Scale (ECAB).

**Results:**

Seventy-three patients were included (male/female ratio = 1.08). Among the 51 patients who used benzodiazepines, 41 (80%) met the criteria for dependence. Nearly half of the patients (49%) reported a history of suicide attempts, with an average of 1.86 attempts (range: 1–4) and a mean interval of 24.9 years since the last episode (range: 2–43). Most attempts (58%) were described as impulsive rather than premeditated.

The most frequently reported motives were negative emotions (22%), family conflicts (19%), delusional ideas (17%), and hallucinations (11%). The most common method was intentional drug ingestion (28%), followed by strangulation (17%) and defenestration (17%); anxiolytics were involved in 60% of drug ingestions.

In 86% of cases, the act occurred when the patient was alone, most often at home (67%), predominantly during nighttime hours (20:00–04:00; 44%) and more frequently in autumn (39%). The suicidal act was mainly reactive in nature (44%).

According to the Ducher Suicide Risk Scale, 40% of patients expressed no death-related thoughts, whereas the remaining 60% presented a range from transient thoughts to active suicidal ideation.

BZD-dependent patients had significantly more frequent histories of suicide attempts (p = 0.01), a shorter duration between the last suicide attempt and the current evaluation (p = 0.03), and more frequent suicidal acts motivated by interpersonal conflicts or psychotic symptoms (delusions, hallucinations) (p = 0.045). They also presented suicidal ideation at the time of the interview (p = 0.045) and had higher scores on the Ducher scale compared to non-dependent patients (p = 0.005).

**Conclusions:**

BZD addiction represents an independent risk factor for suicidality in the elderly. Systematic screening for suicidal risk iscommended in patients with chronic BZD use or dependence.

**Disclosure of Interest:**

None Declared

